# Natural Killer T Cells in Advanced Melanoma Patients Treated with Tremelimumab

**DOI:** 10.1371/journal.pone.0076829

**Published:** 2013-10-22

**Authors:** F. Javier Ibarrondo, Otto O. Yang, Thinle Chodon, Earl Avramis, Yohan Lee, Hooman Sazegar, Jason Jalil, Bartosz Chmielowski, Richard C. Koya, Ingrid Schmid, Jesus Gomez-Navarro, Beth D. Jamieson, Antoni Ribas, Begoña Comin-Anduix

**Affiliations:** 1 Department of Medicine, David Geffen School of Medicine at University of California Los Angeles, Los Angeles, California, United States of America; 2 UCLA AIDS Institute, David Geffen School of Medicine at University of California Los Angeles, Los Angeles, California, United States of America; 3 Department of Microbiology, Immunology, and Molecular Genetics, David Geffen School of Medicine at University of California Los Angeles, Los Angeles, California, United States of America; 4 Department of Medicine, Division of Hematology/Oncology, David Geffen School of Medicine at UCLA, Los Angeles, California, United States of America; 5 Department of Child Psychiatry Branch, NIH/NIMH, Bethesda, Maryland, Untied States of America; 6 Department of Clinical Research, Pfizer Global Research and Development (PGRD), New London, Connecticut, United States of America; 7 Jonsson Comprehensive Cancer Center, University of California Los Angeles, Los Angeles, California, United States of America; 8 Department of Surgery, Division of Surgical Oncology, Department of Medicine, University of California Los Angeles, Los Angeles, California, United States of America; University of Michigan School of Medicine, United States of America

## Abstract

A significant barrier to effective immune clearance of cancer is loss of antitumor cytotoxic T cell activity. Antibodies to block pro-apoptotic/downmodulatory signals to T cells are currently being tested. Because invariant natural killer T cells (iNKT) can regulate the balance of Th1/Th2 cellular immune responses, we characterized the frequencies of circulating iNKT cell subsets in 21 patients with melanoma who received the anti-CTLA4 monoclonal antibody tremelimumab alone and 8 patients who received the antibody in combination with MART-1_26–35_ peptide-pulsed dendritic cells (MART-1/DC). Blood T cell phenotypes and functionality were characterized by flow cytometry before and after treatment. iNKT cells exhibited the central memory phenotype and showed polyfunctional cytokine production. In the combination treatment group, high frequencies of pro-inflammatory Th1 iNKT CD8^+^ cells correlated with positive clinical responses. These results indicate that iNKT cells play a critical role in regulating effective antitumor T cell activity.

## Introduction

Invariant natural killer T cells (Type I NKT cells or iNKT) are a subset of T cells that express a restricted repertoire of T-cell receptors (TCR); in humans the iNKT TCR alpha chain presents a Vα24-JαQ rearrangement that preferentially pairs with a semi-invariant Vβ11 β-chain [1]. The iNKT TCR recognizes glycolipid antigens presented by CD1d, a major histocompatibility complex-like molecule present on the surface of antigen-presenting cells, and that is highly expressed by myeloid dendritic cells (mDCs) [2]–[4]. iNKT cells are actively recruited to infection sites, where they respond to cytokines and interact with CD1d^+^ mDC [5]. In response to stimuli, iNKT cells can release large amounts of regulatory cytokines and are believed to play a pivotal role in the determination of innate and adaptive immune system responses [6]. iNKT cells can be subdivided into three subsets: CD4^+^, CD8^+^ and CD4^−/^CD8^−^ double negative (DN). The CD4^+^ subset has a Th0 profile, being able to produce Th2 and Th1 cytokines such as interleukin 4 (IL-4) and interferon gamma (IFN-γ). DN iNKT cells produce large amounts of Th1 cytokines such as INF-γ and tumor necrosis factor alpha (TNF-α), up-regulate perforin, and release low levels of Th2 cytokines in response to stimuli [7]. Finally, CD8^+^ iNKT cells constitute a Th1-only subset [7], [8]. The balance of CD4^+^ versus DN and/or iNKT CD8^+^ iNKT cells is thought to be critical for proper modulation of immune responses to control inflammatory processes, auto-immunity, and immune surveillance of cancer [7], [9], [10].

The pivotal role of iNKT cells in the regulation of the immune response makes them an attractive target for immunotherapy: the frequency and functionality of iNKT cells is frequently altered in patients with malignancies, autoimmune disorders, and viral infections [11], [12]. Blood iNKT cell frequencies fall in melanoma patients treated with radiation therapy [13], [14] and a drastic reduction in the frequency of iNKT cells capable of secreting IFN-γ has been observed in patients with advanced prostate cancer [15]. Also, the iNKT number has been shown to increase in cancer patients who responded successfully to non-immunological therapies and the number and function of iNKT have been used as prognostic markers in colonorectal, breast, renal cell carcinoma, lung, and melanoma cancers [16], [17]. The specific iNKT cell activator, 9α-galactosyl ceramide (α-GalCer) is being utilized as adjuvant in therapeutic vaccines therapies, and loaded onto dendritic cells (DC) exhibited antitumor cytotoxicity against non-small cell lung cancer [18]–[20]. As a consequence, the role of iNKT cells in immune control and anti-tumor strategies is a rapidly expanding field and is yielding promising results in the elaboration of effective clinical protocols against cancer [21]–[23].

Dendritic cell (DC) vaccines have been tested in multiple clinical trials for the treatment of patients with advanced melanoma [24], [25]. In the context of the interactions between antigen-presenting cells (APC) and T cells, the initiation and maintenance of T cell responses are critically regulated by co-stimulatory and co-inhibitory signaling within the immunological synapse: resting T cells recognize the co-stimulatory molecules CD80 (B7.1) and CD86 (B7.2) presented by DC through the activating co-receptor CD28. Upon activation, T cells upregulate the co-inhibitory receptor CTLA4 (CD152). The interaction of CTLA4 on T cells and B7 co-stimulatory molecules presented by DC inhibits TCR signaling, IL-2 gene transcription and T-cell proliferation [26]. The use of CTLA4-specific human monoclonal antibodies like tremelimumab or ipilimumab to block the interaction between CTLA4 and B7 in order to increase T cell activation has been extensively utilized in clinical trials for patients with cancer, and the administration of tremelimumab to patients with metastatic melanoma has consistently induced objective tumor regression in approximately 10% of patients. Most of these responses are extremely durable, some exceeding seven years [27]–[31]. Despite years of clinical trials utilizing anti-CTLA4 antibodies and dendritic cells, we still do not know the immune parameters that correlate with positive clinical responses to these treatments; uncovering such parameters would be invaluable for predicting the prognosis of patients undergoing immunotherapy.

Since iNKT cells are a fundamental link between dendritic cells and the adaptive immune system, we hypothesized that iNKT cells may be implicated in the responses to CTLA4 blocking monoclonal antibodies, in particular when administered together with a DC vaccine. Here we studied the changes in the iNKT cell populations in blood from patients treated with tremelimumab as a single agent or in combination with DC vaccination. Our results demonstrate that iNKT cells may play a role in the mechanism of action of CTLA4 blocking antibodies when combined with a DC vaccine and that high CD8^+^ iNKT frequency correlates with positive clinical outcome.

## Materials and Methods

### Clinical Trials and Study Samples

**Figure 1:**
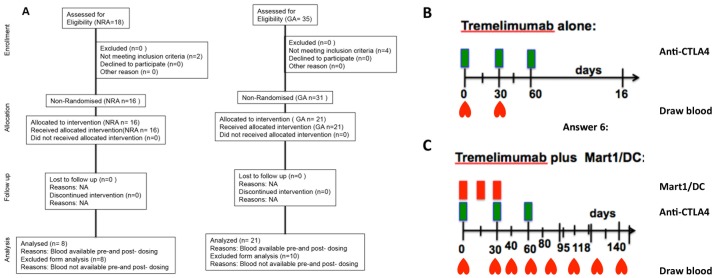
Description of the two tremelimumab clinical trials. (A) CONSORT flowchart. Tremelimumab alone (GA). (B) and tremelimumab plus Mart1/DC (NRA). (C). Baseline is day 0, and numbers represent days after dosing that blood was drawn. NA-Not Aplicable.

Peripheral blood samples were obtained from 29 patients with stage IIIc or IV melanoma treated at UCLA within two independent clinical trials based on the administration of tremelimumab (Pfizer, New London, CT). Written informed consent was obtained for both trials [34]. Eight patients were treated within a phase I clinical trial (from a total of sixteen) of a dose escalation of intravenous infusion of tremelimumab (3–15 mg/kg) administered concomitantly with three fixed doses of 1×10^7^ autologous DC pulsed with the MART-1_26–35_ immunodominant peptide epitope (MART-1/DC) manufactured as previously described (University of California Los Angeles Institutional Review Board number (UCLA IRB#) 03-12-023, IND# 11579, clinical trial registration number NCT0090896) [32], [33], [34]. The study denomination of NRA and a patient-specific number was assigned to these patients. The remaining 21 patients participated in a phase II clinical trial of single agent tremelimumab administered at 15 mg/kg every 3 months ((UCLA IRB)# 06-06-093, IND# 100453, clinical trial registration number NCT00471887) [33], [34] (Figure 1). The study denomination GA and a patient-specific number were assigned. A modified Response Evaluation Criteria in Solid Tumors (RECIST, [32 to 35]) was used to allocate an objective clinical response, where skin and subcutaneous lesions evaluable only by physical exam were considered measurable if adequately recorded using a photographic camera with a measuring tape or ruler; there was no minimum size restriction for these lesions. A progressive disease–at least a 20% increase in the sum of the longest diameter of target lesions, taking as reference the smallest sum longest diameter recorded since the treatment started or the appearance of one or more new lesions; partial response–at least a 30% decrease in the sum of the longest diameter of target lesions, taking as reference the baseline sum longest diameter; stable disease–neither sufficient shrinkage to qualify for partial response nor sufficient increase to qualify for progressive disease, taking as reference the smallest sum longest diameter since the treatment started; and complete response–the disappearance of all target lesions [32]–[35]. Those categories were used to classify the patients as responders (PR, CR) or non-responders (PD, SD).

### Sample Procurement and Processing

Pre-treatment leukapheresis and phlebotomies were carried out for all the participants in both studies. The post-treatment leukapheresis was performed one month after treatment for the single agent tremelimumab trial (GA participants, median 38 days post-treatment). In order to compare the two study treatments, we chose a similar time point for both (Figure 1b), approximately one month after treatment initiations. In all cases, a baseline sample taken prior to any treatment served as control. In addition to the post-dosing leukapheresis, NRA patients had post-treatment peripheral blood samples taken around days: 31, 43, 60, 80, 95 and 118 during study participation. PBMC were isolated by Ficoll-Hypaque (Amersham Pharmacia, Piscataway, NJ) gradient centrifugation and cryopreserved in liquid nitrogen in RPMI (Gibco-BRL, Gaithersburg, MD) supplemented with 20% (all percentages represent v/v) heat-inactivated human AB serum (Omega Scientific, Tarzana, CA) and 10% dimethylsulfoxide (DMSO, Sigma, St. Louis, MO). Phenotype analyses were run using aliquots of cryopreserved PBMC thawed and immediately diluted with RPMI complete media consisting of 10% human AB serum, and 1% penicillin, streptomycin and amphotericin (Omega Scientific). Cells were washed and subjected to enzymatic treatment with DNase (0.002%, Sigma) for 1 hour at 37°C. After removing the DNase, PBMC were kept overnight in the incubator at 37°C and 5%CO_2_ and stained for flow cytometry the next morning.

### Flow Cytometry Staining

Surface Staining: After resting, PBMC were centrifuged (468 g), resuspended in 100 µl of adult bovine serum (Omega Scientific) and stained for flow cytometry for 15 minutes at room temperature as described in [32]. The reaction was stopped by adding 3 ml of phosphate buffered saline without calcium and magnesium (PBS) (Lonza, MD, USA). After centrifugation, the pellet was resuspended in PBS, and the dead cell discriminator 7AAD was added to the PBMC. All cocktail combinations of pre-conjugated antibodies were used at saturating conditions. (See Table S2).

For intracellular cytokine staining (ICS), after resting overnight, PBMCs were surface stained only for CD107a as described above. Then, a concentration of 1.5 million per ml of PBMC were pulsed with or without a final concentration of 300 IU/ml IL2 plus 50 ng/ml anti-CD3 (OKT-3; Centocor Ortho Biotech Inc., Horsham, PA) [36] for six hours. After the first hour of incubation, 1 µl of BDGolgiPlug (BDbioscience, San Jose, CA) was added. After the stimulation, cells were put on ice for 5 minutes. Then, surface staining for CD3, CD4, CD8, iNKT, and CD107a was performed. After washing the excess of antibodies, cells were fixed (BD Cytofix/Cytoperm), permeabilized (BD Perm/Wash Buffer), and stained for IFNμ, IL4, and IL10 following the manufacturer’s instructions. All cocktail combinations of pre-conjugated antibodies were used at saturating conditions (See Table S3). A combination of anti-mouse IgG Negative Control Compensation Particles (BD Biosciences) and PBMC were used for compensation purposes. The fluorescence- minus-one (FMO) approach was used to gate appropriately [32], [58]. We acquired between 500,000 and one million lymphocytes with a LSR II Flow Cytometer (BD Biosciences). All flow data analyses were done with the FlowJo software (Tree Star Inc., Asland, OR). The gating strategy was displayed in (Figure S1a).

The absolute numbers of cells in the samples were monitored by flow cytometry utilizing BD Trucount™ kits (BD Biosciences) as indicated by the manufacturer.

iNKT was defined as double positive for TCR-Vβ11 and TCR-Vα24.

### Statistical Analysis

The statistical analyses are mainly descriptive because of the small size of the population. Consequently, descriptive statistical analyses were carried out with Minitab® Statistical Software (State College, PA) and GraphPad Prism (GraphPad, San Diego, CA). In all cases we collected between half and one million lymphocytes through flow cytometry. Wilcoxon Sign Rank Test was used to compare pre- and post-treatment tremelimumab effect. Mann Whitney test was utilized to compare the analysis of results from responders versus non-responders.

## Results

### Patient Characteristics, Response and Toxicities

21 patients received tremelimumab as a single agent (coded as GA) and eight patients received tremelimumab together with MART-1/DC (coded as NRA; Figure 1b and c). Table S1 shows the baseline patient characteristics, the treatment received and the clinical outcome. 14 of 27 patients (52%) had M1c metastatic melanoma with visceral metastasis and/or high lactate dehydrogenase (LDH). Overall, 7 patients (3 in the tremelimumab single agent, and 4 in the tremelimumab plus MART-1/DC protocol) had objective positive responses to the treatment, resulting in sustained and durable tumor regression in 6 individuals. These 7 patients had either stage IIIc or M1a metastatic melanoma. Toxicities were described previously [32]–[34].

### Effects of Tremelimumab on T and iNKT Cell Concentrations

**Figure 2:**
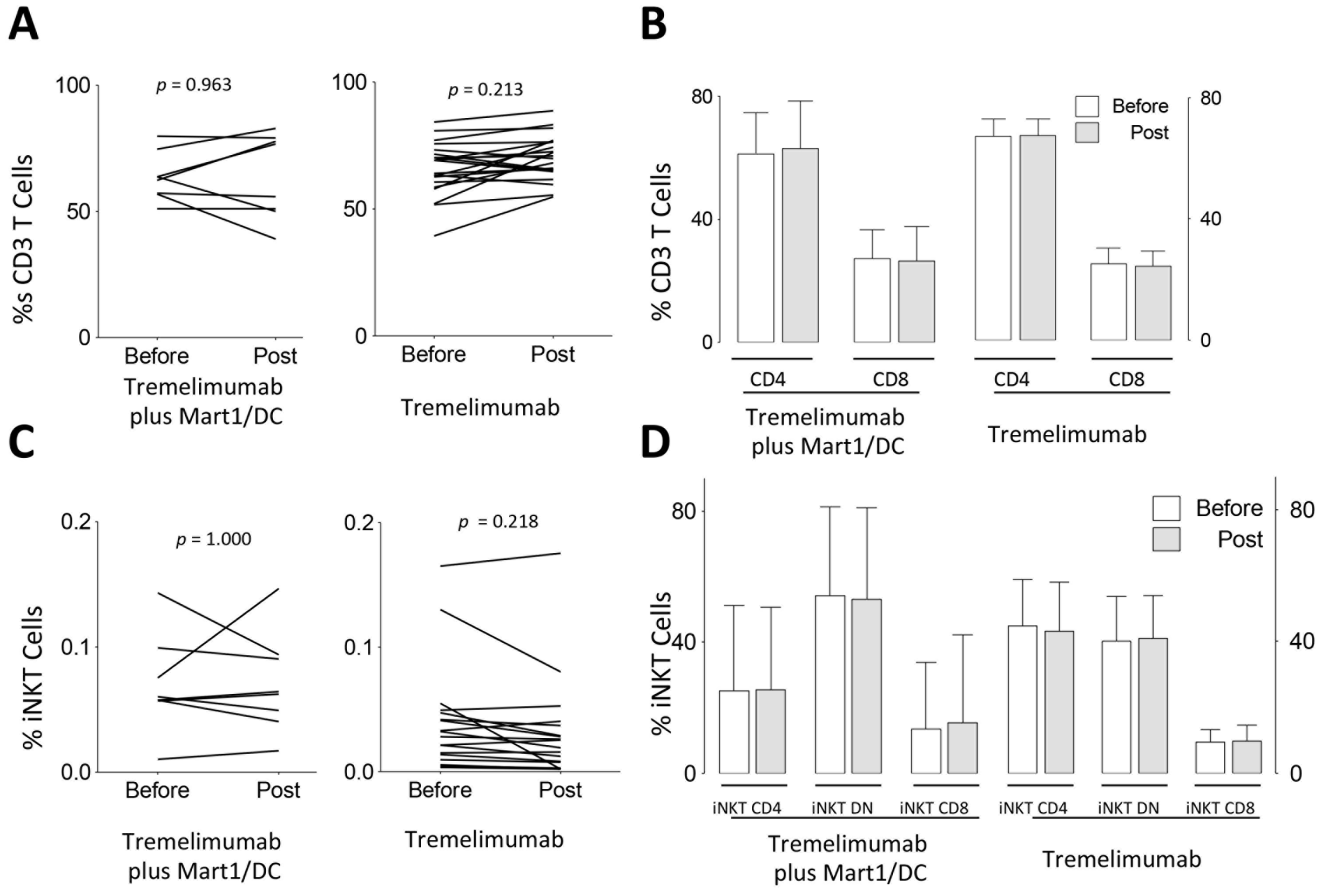
CD3 and iNKT cells and subsets before and after tremelimumab single agent treatment. After gating by morphology, live cells were gated on CD3^+^ T cells; CD3^+^ T cells (A), CD4^+^ and CD8^+^ (B); iNKT cells (double positive for TCR-Vα24/Vβ11; C); iNKT CD4^+^, iNKT CD8^+^ and iNKT DN (D) subsets were analyzed before (open boxes in B and D panels) and after treatment (greyed boxes in B and D panels). In panels b and d, results are presented as average of the percentage and standard deviation (sd) for both clinical trials (n = 21 for tremelimumab alone and n = 8 for tremelimumab plus Mart1/DC).

In both clinical trials, we found a non-significant increase in the percentage of CD3^+^ T cells after dosing (Figure 2A). On the other hand, none of the treatments had any effect on the percentages of CD4^+^ or CD8^+^ T cells (Figure 2B).

The proportion of circulating iNKT cells was not affected by the treatments, (Figure 2C right panel). Incidentally, we measured slightly higher frequencies of iNKT with a Th1 profile (iNKT DN and iNKT CD8^+^ cells) at baseline in the tremelimumab plus MART-1/DC study (p = 0.1796 and p = 0.6084 for iNKT DN and iNKT CD8^+^ respectively; Figure 2D) although Fisher’s exact test for high iNKT CD8^+^ percentages (above 15%) showed no difference between both clinical trials at baseline (p = 0.5968).

### Correlation of iNKT Cell Subsets to Clinical Response in Combination Treatment

**Figure 3:**
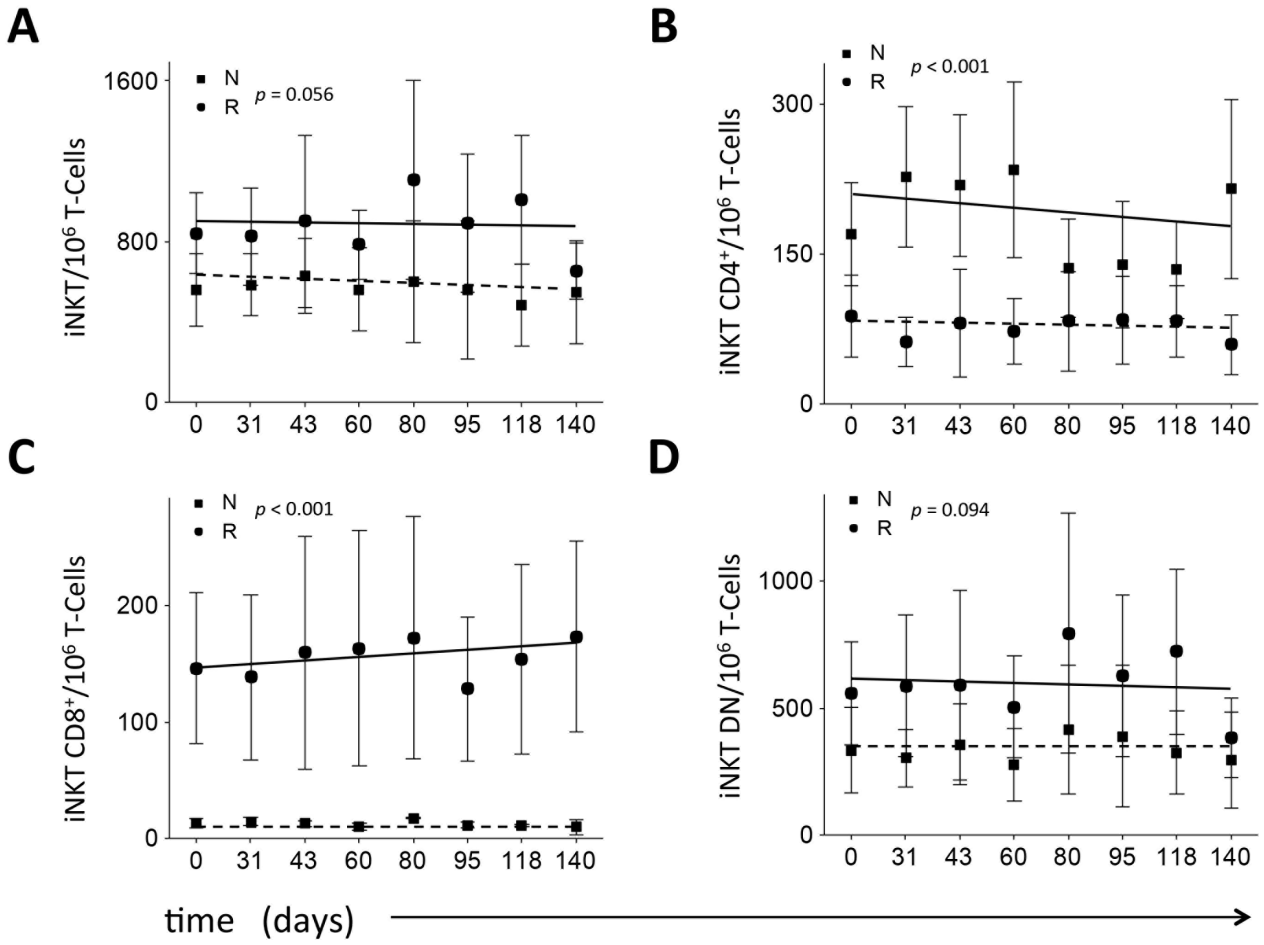
Percentage of iNKT cells and its subsets in samples taken from patients in the tremelimumab plus MART-1/DC study. Evolution of iNKT cells and iNKT cells subsets during the treatment in the combined study in responders (circles) and non-responders (squares); (A) number of iNKT per million T cells; (B) number of iNKT CD4^+^ per million T-cells; (C) number of iNKT CD8^+^ per million T-cells and; (D) number of iNKT DN per million T-cells. In the Figure, regression lines for each data set are shown; error bars represent one sd from the mean for each point.

When we compared patient responses to the treatment of tremelimumab plus MART-1/DC, we found that the individuals with a positive clinical outcome (responders) showed higher total numbers of iNKT cells than individuals who did not respond to the treatment (non-responders) at all the tested time points, including baseline (p = 0.056; Figure?>;3A). The iNKT cell frequencies remained unchanged in both groups after treatment (Figure 3A). Further characterization of iNKT cell subsets revealed that although the relative proportions of the different iNKT subsets within each group were not modified by the treatment, the total number of CD4^+^ iNKT cells in responders was lower than in non-responders (mean 77±71 cells/million T cells versus 189±120 cells/million T cells in non-responders; p<0.001; Figure 3B). We also found a higher number of CD8^+^ iNKT cells in responders (154±150 cells/million T cells, versus 13±6 cells/million T cells in non-responders; p<0.001; Figure 3C). The total number of DN iNKT cells was also higher in responders, although the difference was not significant (596±553 cells/million T cells versus 334±224 cells/million T cells; p = 0.094; Figure 3D). We also found that the percentage of total CD4^+^ T cells was slightly higher in responders than in non-responders (p<0.001; Figure S2A).

### Phenotypes of iNKT Cells in Tremelimumab-Treated Patients

**Figure 4:**
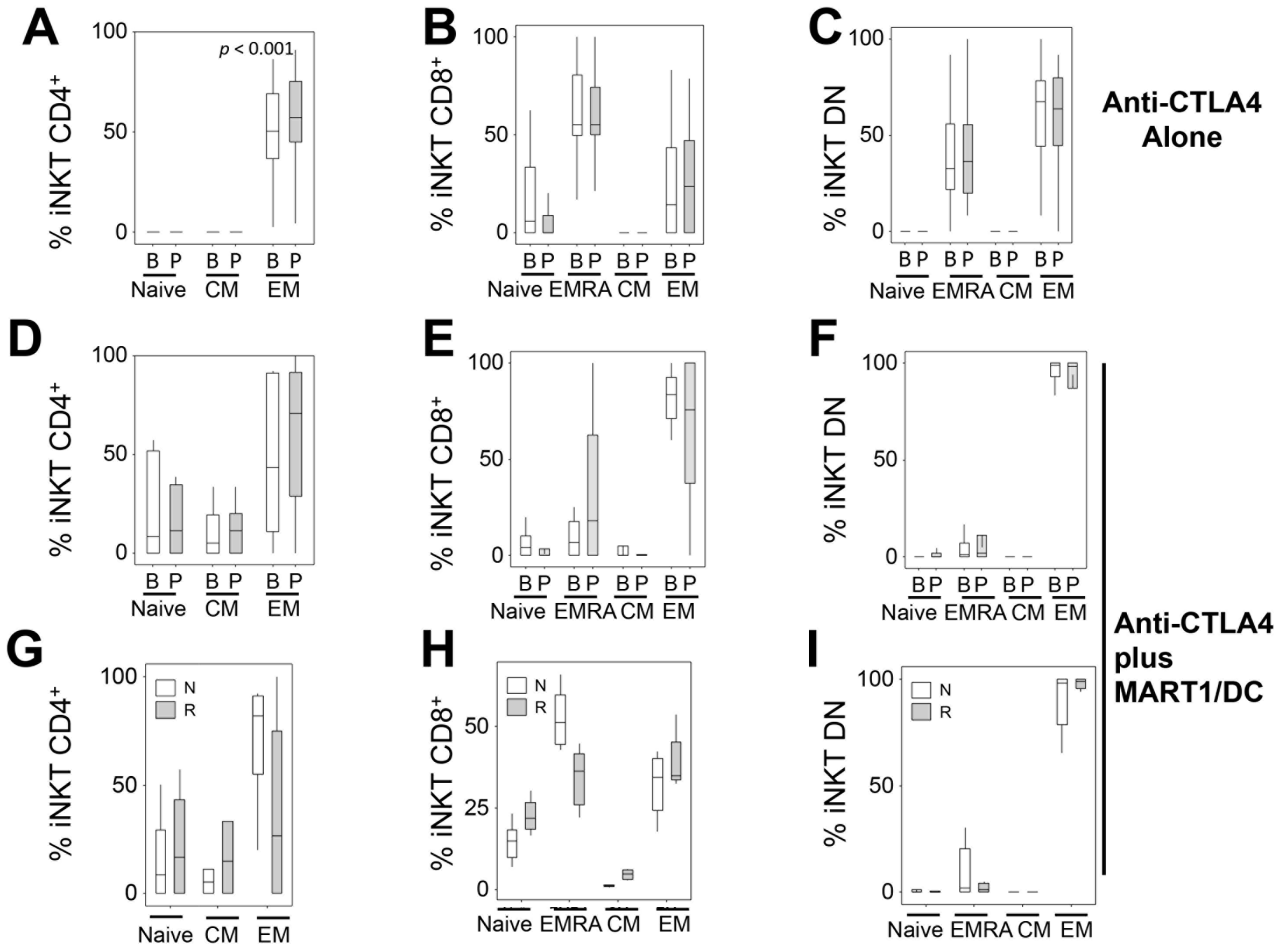
Phenotyping of iNKT subsets. Frequencies of iNKT CD4 (A, D, G), iNKT CD8 (B, E, H), and DN (C, F, I) subsets. Upper panels (A, B, C), tremelimumab alone clinical trial; middle and lower panels (D, E, F), tremelimumab plus Mart-1/DC trial. First and second row represented all iNKT subsets analyzed for CCR7 and CD45RA expression before (B) and after (P) treatment for both clinical trials. The last row represented iNKT subsets analyzed for CCR7 and CD45RA expression for responders (R) and non-responders (N) only for tremelimumab plus Mart-1/DC trial. EMRA = effector memory RA; CM = central memory, and EM = effector memory.

We analyzed the maturation status of the different T-cell populations by flow cytometry defining naïve (N, CCR7^+^CD45RA^+^), central memory (CM, CCR7^+^CD45RA^−^), effector memory (EM, CCR7^−^CD45RA^−^), and effector memory-RA (EMRA, CCR7^−^CD45RA^+^) T-cells [37]. We observed that the majority of CD4^+^ iNKT cells had a CCR7^−^CD45RA^−^ EM phenotype (n = 21, median 50.25%), and the proportion of this subpopulation was increased after treatment (median 57.21%; p<0.01 compared to baseline, Figure 4A). The CD8^+^ iNKT cell subset showed a non-significant increase, while the DN iNKT cell subset showed a non-significant decrease of the EM phenotype percentage after treatment (Figure 4B, C). DN and CD8^+^ iNKT subsets, but not the CD4^+^ subset, presented sizable subpopulations with the EMRA CCR7^−^CD45RA^+^ phenotype whose frequencies were unchanged by tremelimumab (Figure. 4B, C). Finally, a substantial fraction of CD8^+^ iNKT cells showed the naïve phenotype, which was non-significantly reduced after dosing with tremelimumab (Figure 4B).

### Phenotype of iNKT Cells in Tremelimumab plus MART-1/DC-Treated Patients

In patients treated with tremelimumab plus MART-1/DC, we found the same profile in the maturation status of the three iNKT subsets as seen in the tremelimumab-alone treatment with minor differences: there were small populations of naïve and central memory phenotypes in CD4^+^ iNKT cells (Figure 4D) and lower frequencies of EMRA phenotype in DN and CD8^+^ iNKT cells compared to the tremelimumab-alone treatment (Figure 4E, F). The responder/non responder segregation presented a higher percentage of naïve, CM CD4+iNKT and CD8+iNKT in responders (Figure 4G, H), and scarcity of DN iNKT (Figure 4F). On the contrary, the percentage of EM subset was lower in CD4+iNKT and CD8+iNKT of the responders (Figure 4G, H). The same high percentage of EM was detected in DN iNKT independent of treatment and response status. (Figure 4F, I).

**Figure 5:**
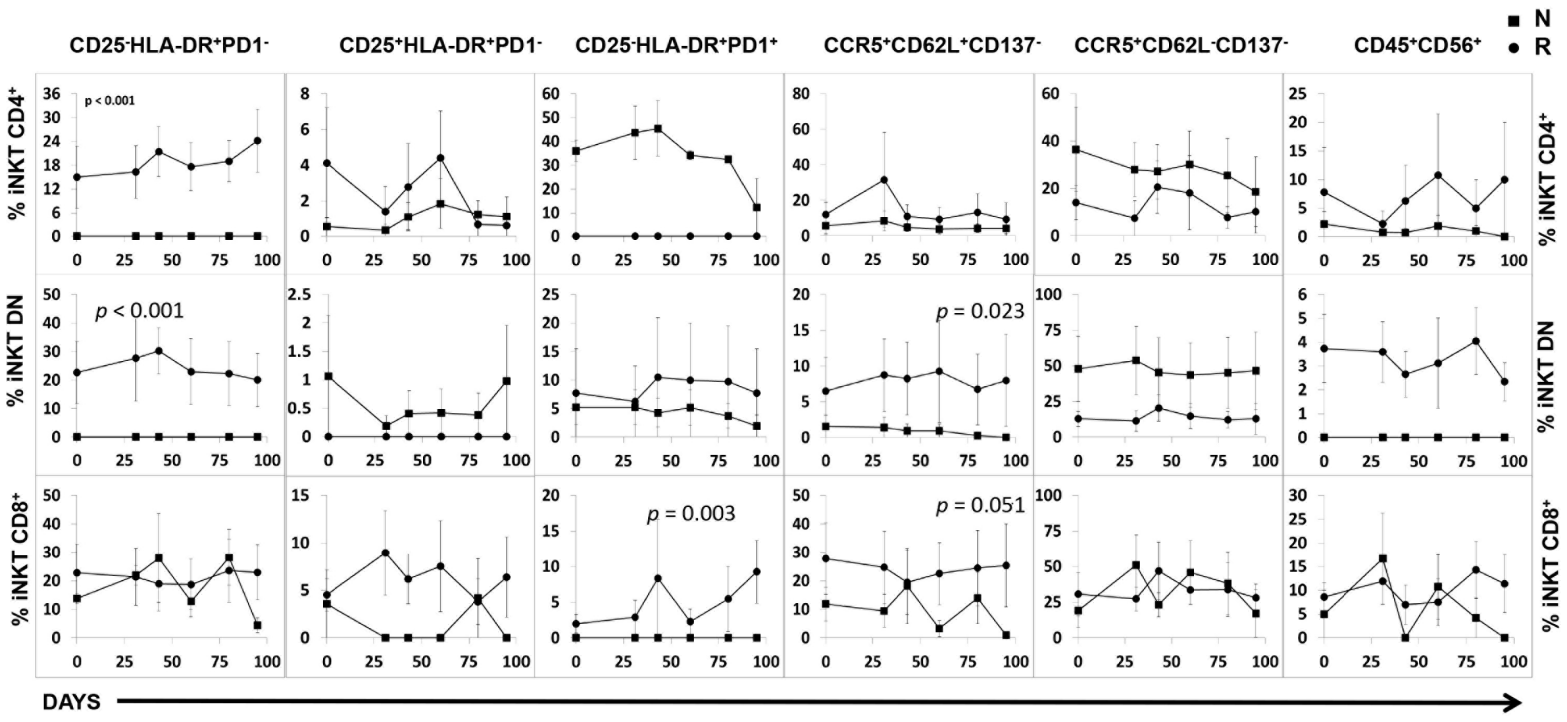
Phenotype analysis of iNKT cells over time after tremelimumab plus MART-1/DC. Analysis of early activated CD25−HLA−DR+PD1− (A, B, C) and CD25+HLA−DR+PD1− (D, E, F), late activated CD25−HLA−DR+PD1+ (G, H, I), central memory CCR5+CD62L+CD137− (J, K, L), effector CCR5+CD62L−CD137− (M, N, O), and terminal differentiated CD45RA+CD56+ (P, R, S) iNKT cells. The percentages of iNKT CD4+ (A, D, G, J, M, P), iNKT DN (B, E, H, K, N, R) and iNKT CD8+ (C, F, I, L, O, S) cells were quantified at different times during the double treatment with tremelimumab plus MART-1/DC. Results expressed as means plus/minus sd and statistical significance. Squares represent non-responders and circles responders to the treatment.

To further characterize the iNKT cells from the tremelimumab plus Mart1/DC trial, which presented a higher rate of positive clinical responders (50%, four of eight) than the single agent clinical trial (14%, three of twenty one), PBMC were evaluated by multicolor phenotyping utilizing two additional panels. This strategy enabled simultaneous analysis of additional activation markers at each time point on all the iNKT cells subsets. Although the percentage of all iNKT activation-based phenotypes remained unchanged during and after treatment (Figure 5), we uncovered different distributions of some phenotypes depending on the clinical response (Table 1).

**Table 1:** Phenotyping characteristic of Tremelimumab plus MART1/DC patients segregated by responders (R) and non-responders (NR) independent of the time points.

		Early Activated (%)	Late Activated (%)	EFFECTORS (%)	EFFECTORS Terminally differentiated (%)
CD4^+^ -iNKT	R	9.83±1.48	0.10±0.11	13.00±3.23	7.26±2.42
	NR	1.30±0.27	36.82±2.72	23.53±6.71	1.72±0.49
DN-iNKT	R	5.47±1.53	8.94±3.31	13.30±2.66	3.06±0.42
	NR	0.18±0.06	5.76±1.10	37.57±8.83	0.21±0.09
CD8^+^-iNKT	R	11.08±1.57	0.04±0.03	30.82±4.36	10.83±1.58
	NR	7.92±1.96	0.01±0.00	32.14±6.50	6.62±2.17

Early activated (CD25^−^HLA−DR^+^PD1^−^ & CD25^+^HLA−DR^+^PD1^−^), n = 40 (NR) to 48 (R) time points; late activated (CD25^−^HLA−DR^+^PD1^+^), n = 20 (NR) to 31 (R); effectors (CCR5^+^CD62L^+^CD137^−^), n = 20 (NR) to 31 (R); and effectors terminally differentiated (CD45^+^CD56^+^), n = 20 (NR) to 31 (R).

We utilized CD25 and HLA-DR plus PD1 staining to define early (PD1^−^: CD25^−^HLA−DR^+^PD1^−^ and CD25^+^HLA-DR^+^PD1^−^) and late (PD1^+^: CD25^−^HLA−DR^+^PD1^+^) activated T-cells. In all of the iNKT subsets, a small increase of the early activated phenotype was found in clinical responders (Table 1, and Figure 5A to F).

The frequency of the late activated phenotype was only present in the CD4+ iNKT cells of the responders (36.82±2.72) (Figure 5G), but absent for CD8^+^ iNKT cells (Figure 5L). The expression of the late activated phenotype in DN iNKT cells was low but equally represented in responders and non-responders (8.94±3.31% versus 5.76±1.10%) (Table 1, and Figure 5I).

A second panel was established to examine central memory (CCR5^+^CD62L^+^CD137^−^), and effector (CCR5^+^CD62L^−^CD137^−^) T cell phenotypes [38]–[40].

The central memory phenotype (CCR5^+^CD62L^+^CD137^−^) was exhibited by all iNKT cells subsets (Figure 5J, K, L) and significantly more frequent in Th1 iNKT cells from responders (p = 0.023 and p = 0.051 for DN and CD8^+^ iNKT cells respectively, Figure 5K, L). We detected comparable expression of the effector phenotype in CD8^+^ iNKT cell in both responders/non-responders (Table 1, and Figure 5O). However, the percentage of effector CD4^+^- and DN- iNKT cells was lower in responders. (Table 1, and Figure 5M & N).

Finally, the expression of CD45RA and CD56 were utilized to confirm the effector phenotypes (terminally differentiated effector T cells are CD45RA^+^CD56^+^ [41]). This phenotype was detected mainly at low expression in all iNKT subsets of responders, and less than ten percent of the CD8+ iNKT non-responders (Table 1, and Figure 5 P, R, S).

Altogether, these data show that although the proportions of different phenotypes vary between the three iNKT subsets, there was no significant difference in their distribution between responders and non-responders.

### Polyfunctionality of T and iNKT Cells in Tremelimumab Plus MART-1/DC Treated Patients

We studied the functionality of T cells [42]–[44] after 95 days of treatment compared to baseline in the tremelimumab plus Mart1/DC protocol. Flow cytometric analysis included expression of interleukin 4 (IL-4, marker for Th2), IFN-γ (Th1), IL-10 (immunosuppressive), and CD107a (lysosomal-associated membrane protein-1, LAMP-1, marker of cytotoxic degranulation). A fold change of two was considered an actual change from baseline.

**Figure 6:**
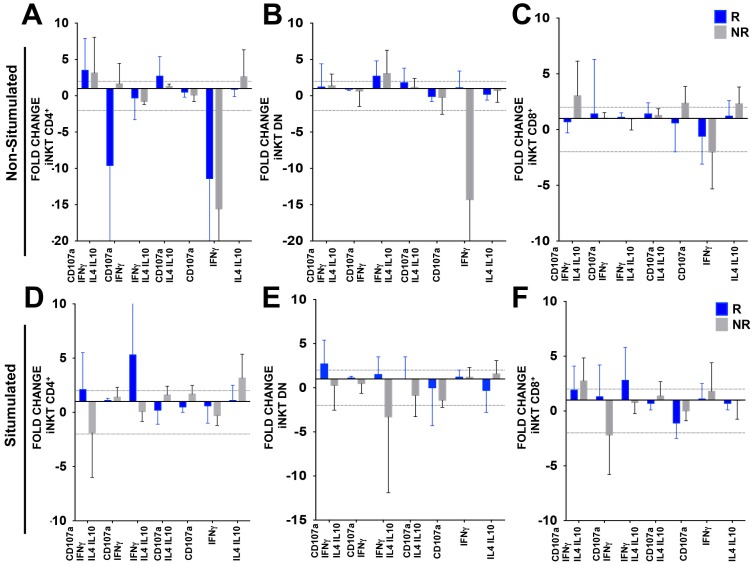
Functionality of iNKT subsets after tremelimumab plus Mart-1/DC treatment. Intracellular staining of IFNγ, IL4, IL10 and CD107a in iNKT cells after tremelimumab plus Mart-1/DC treatment between responders (blue) and non-responders (grey) were measured in PBMC stimulated with OKT-3 plus IL2 for six hours. Y axis displayed fold change of CD4^+^- DN- CD8^+^-iNKT cells after treatment. X axis showed the different cytokines expressed by the cells. Dot line showed two fold change with respect to baseline, and any bar over the two fold is considered a change.

As expected, upon stimulation CD4^+^ and DN iNKT cells exhibited a mixture of cytokine expression (Figure 6). The CD4^+^ iNKT cell subset presented a polyfunctional profile (two or more intracellular markers; Figure 6A, D), mainly after stimulation in responders. Only the non-responders secreted IL4/IL10 alone (Figure 6A, D). DN iNKT cells showed polyfunctionality after stimulation in responders, but not in non-stimulated cells from non-responders; both responders and non-responders expressed a combination of CD107a and IL4/IL10 (Figure 6B, D). CD8^+^ iNKT cells displayed some degree of polyfunctionality in the non-responders, and a co-expression of IFNγ and IL4/IL10 the responders after stimulation (Figure 6C, D).

Total T cells showed the same profile as iNKT cells (Figure S4).

## Discussion

This study was aimed to characterize iNKT cells in patients with metastatic melanoma treated with tremelimumab as single agent or in combination with MART-1/DC. We found that following both treatments, the frequencies of total iNKT cells and their subsets were altered, and that high iNKT CD8^+^ cell frequencies correlated with successful outcomes in the combined treatment. Since we compared the pre- and post-treatment iNKT cell levels, age and sex, which are known to affect iNKT cell frequency [16], [17], were not confounding factors.

The most striking finding emerging from our work is the close correlation between the positive clinical outcome for the tremelimumab plus MART-1/DC treatment and the high frequency of the CD8^+^ iNKT cell subset in the responders, with a corresponding reduction in the frequency of the CD4^+^ iNKT cell subset. This correlation was dependent on the presence of primed dendritic cells, since it was not observed in the antibody-alone treatment. Although some immune parameters in T cells and monocytes have been variably associated with response to the combination regimen [34], [45]; although the small number of patients, this is the first demonstration of a consistent immune marker for treatment response: a high CD8^+^/CD4^+^ iNKT cell ratio. Only one of the four responders in the combined treatment received previous immunotherapy, another received chemotherapy and the remaining two had no prior treatment [44], indicating that previous immunotherapy did not cause the observed response. Because of this, we hypothesize that the combination of high CD8^+^/CD4^+^ iNKT cell ratio, tremelimumab, and the introduction of primed dendritic cells [46] led to the four responses, as both clinical trials were based on tremelimumab and the long term effects of tremelimumab as an anti-CTLA4 blockade antibody are well described [29], [30].

The frequencies for iNKT cells at baseline (0.01% for both study arms) and the frequencies of CD8^+^ iNKT cells were typical of those found in healthy individuals [11]. The CD8^+^ NKT cells are the most variable subset of iNKT cells in humans [47], [48], usually representing between 10% and 15% of all iNKT cells in healthy individuals [7]. In our study, the mean frequencies of iNKT CD8^+^ fell within normal values and the percentages of CD8^+^ iNKT cells above 15% at baseline was indistinguishable between both studies, indicating that high CD8^+^ iNKT cells in responders was in fact related to treatment success.

As expected, we found that iNKT CD4^+^ cells secreted a mixture of Th1 and Th2 cytokines in the same cell. This response was independent of clinical response of the patients. Also, as previously described [47], [48], we found that iNKT CD8^+^ cells produced some IFN-γ and expressed CD107, but did produce IL-4/IL-10. We speculated that the co-expression could be due to IFN-γ with IL10 (not IL4) in response to a Th1 negative feed-back [49] and/or due to the plasticity of iNKT cells [7]. Fold change cytokine production was low in responders, which can be attributed to the maturation status of the tremelimumab plus MART1/DC iNKT cells. Those cells were mainly EM and EMRA of which only approximately 10% were activated. Furthermore, the presence of naïve and CM found on CD4+ and CD8+ iNKT cells did not contribute to cytokine production.

However, in agreement with our results it has been previously described that high frequencies of iNKT cells, and more importantly high frequencies of interferon gamma-producing iNKT cells are correlated with tumor surveillance and increased survival in non-small cell lung cancer patients, and an adjuvant effect iNKT cells appears to be a determinant for tumor eradication [19], [20].

As we found significant positive response linked to high CD8^+^ iNKT cells in the tremelimumab plus MART-1/DC protocol, it is reasonable to hypothesize that interactions between dendritic cells and iNKT [50] cells play a fundamental role in the positive clinical outcome. The dendritic cells that we examined are typically immature dendritic cells [34], and an average of 88% expressed CD1d (data not shown). Given the interaction of iNKT cells with immature dendritic cells and their ability to mediate their maturation through cytokines such as interferon gamma [51], this interaction may be important given that mature dendritic cells are more effective in helping to eliminate tumors [52].

We found that most iNKT cells express CCR5, CD45RA HLA-DR and PD1, reflecting activated central memory and effector memory phenotypes, in agreement with previous studies [11], [53]–[55]. Although none of the treatments affected the expression of iNKT cell phenotypes or cytokine secretion, we observed segregation of the activation markers when we compared subjects that responded to the combined treatment with non-responders. CD25, HLA-DR, CCR5, and CD45RA showed higher expression in responders, while PD1 was over-represented in non-responders. These results reinforce the idea that higher T cell activation correlates with better cancer control [56]–[58]. Furthermore, the finding that responders to combination treatment had this phenotype before treatment suggests that they may be useful predictors of clinical response. It is yet unclear whether strategies using iNKT cells as adjuvants in immunotherapy approaches via administration of alpha-galactosyl ceramide to expand iNKT cells or differentiation of iNKT cells from pluripotent or embryonic cells [59]–[61] will be effective.

In conclusion, it is becoming evident that iNKT cells can play a pivotal role in anti-cancer immunity. We have characterized peripheral iNKT cells in two different tremelimumab clinical trials for patients with advanced melanoma, finding that responders to tremelimumab plus Mart1/DC treatment had higher frequency of the Th1 type CD8^+^ iNKT cells and a lower frequency Th2/anti-inflammatory CD4^+^ iNKT cells than non-responders. Further studies will be required to confirm that the CD8^+^/CD4^+^ iNKT cell ratio predicts a positive outcome in dendritic cell cancer immunotherapies, and whether such therapies would benefit from adjuvant protocols to selectively drive activation and proliferation of CD8^+^iNKT cells.

## Supporting Information

Figure S1 **Gating strategy.** (A) Surface staining. Live cells (7AAD^−^) were gated on morphology (Lymphocytes & monocytes) based on side and forward light scattering. 7AAD^−/^CD3^+^ T cells were separated into three subsets. CD4^+^, CD8^+^, and iNKT (double positive for TCR-Vα24/Vβ11). iNKT were further separated into iNKT-CD8^+^; iNKT CD4^+,^and iNKT-DN (double negative CD8^−/^CD4^−^). These T-cell subsets were then examined for different activation markers, in this example: CD25 & HLA-DR (Activation markers), and PD1 (exhausted cells). Bolean gates were applied to these subsets. (B) ICS. After doublet elimination, lymphocytes were gated on morphology, based on scatter. CD3^+^ T cells were separated into three subsets: CD4^+^, CD8^+^, and iNKT (double positive for TCR-Vα24/Vβ11). iNKT were further separated into iNKT-CD8^+^; iNKT CD4^+,^and iNKT-DN (double negative CD8^−/^CD4^−^). These T-cell subsets were then examined for CD107a, interferon-gammma (IFNγ), CD107a, and a combination of IL4/IL10.
(TIF)

Figure S2 **CD3 T cells after tremelimumab exposure.** Percentage of CD3^+^CD4^+^ (light gray) and CD3^+^CD8^+^ (dark gray) T cells before (B) and at different time-points of CTLA4 dosing in the tremelimumab as single agent treatment (***p<0.001).
(TIF)

Figure S3 **Functionality of T subsets after tremelimumab plus Mart-1/DC treatment.** Intracellular staining of IFNγ, IL4, IL10 and CD107a in iNKT cells after tremelimumab plus Mart-1/DC treatment between responders (blue) and non-responders (grey) were measured in PBMC stimulated with OKT-3 plus IL2 for six hours. Y axis displayed fold change of CD4^+^- and CD8^+^- T cells after treatment. X axis showed the different cytokines expressed by the cells. Dot line showed two fold change with respect to baseline, and any bar over the two fold is considered a change.
(TIF)

Table S1 **Patient characteristics.**
(DOCX)

Table S2 **Antibody combinations for multicolor surface immune phenotyping of NRA and GA patients.**
(PDF)

Table S3 **Antibody combinations for ICS of NRA patients. In parenthesis the clone used.**
(PDF)
