# Supplementary figures and images for: Natural Killer T Cells in Advanced Melanoma Patients Treated with Tremelimumab
Source: PLoS One. 2013 Oct 22;8(10):e76829. doi: 10.1371/journal.pone.0076829 (PMC3805549; doi:10.1371/journal.pone.0076829)

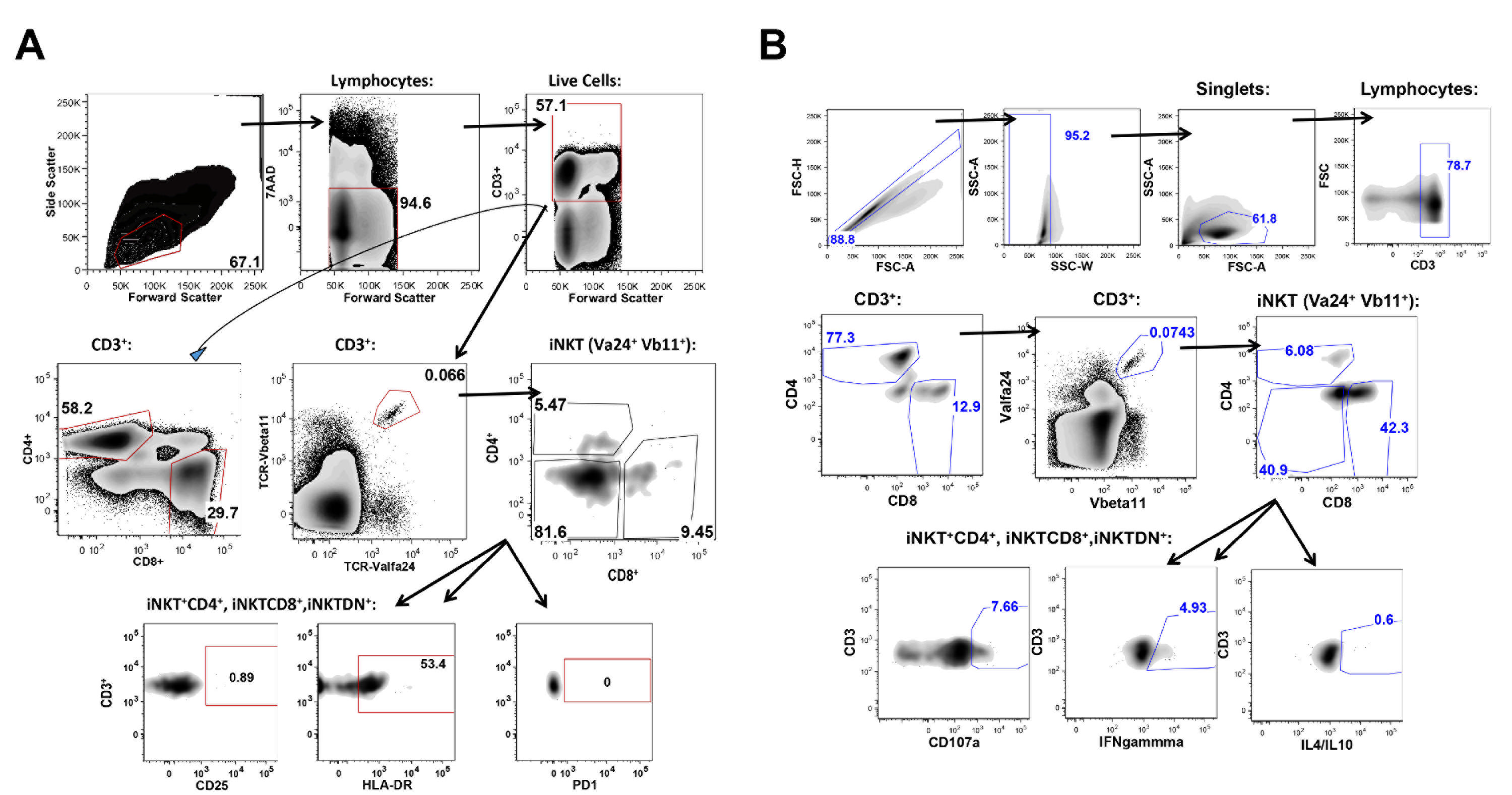

Supplement: Figure S1 — Gating strategy. (A) Surface staining. Live cells (7AAD−) were gated on morphology (Lymphocytes & monocytes) based on side and forward light scattering. 7AAD−/CD3+ T cells were separated into three subsets. CD4+, CD8+, and iNKT (double positive for TCR-Vα24/Vβ11). iNKT were further separated into iNKT-CD8+; iNKT CD4+,and iNKT-DN (double negative CD8−/CD4−). These T-cell subsets were then examined for different activation markers, in this example: CD25 & HLA-DR (Activation markers), and PD1 (exhausted cells). Bolean gates were applied to these subsets. (B) ICS. After doublet elimination, lymphocytes were gated on morphology, based on scatter. CD3+ T cells were separated into three subsets: CD4+, CD8+, and iNKT (double positive for TCR-Vα24/Vβ11). iNKT were further separated into iNKT-CD8+; iNKT CD4+,and iNKT-DN (double negative CD8−/CD4−). These T-cell subsets were then examined for CD107a, interferon-gammma (IFNγ), CD107a, and a combination of IL4/IL10. (TIF) [file pone.0076829.s001.tif]

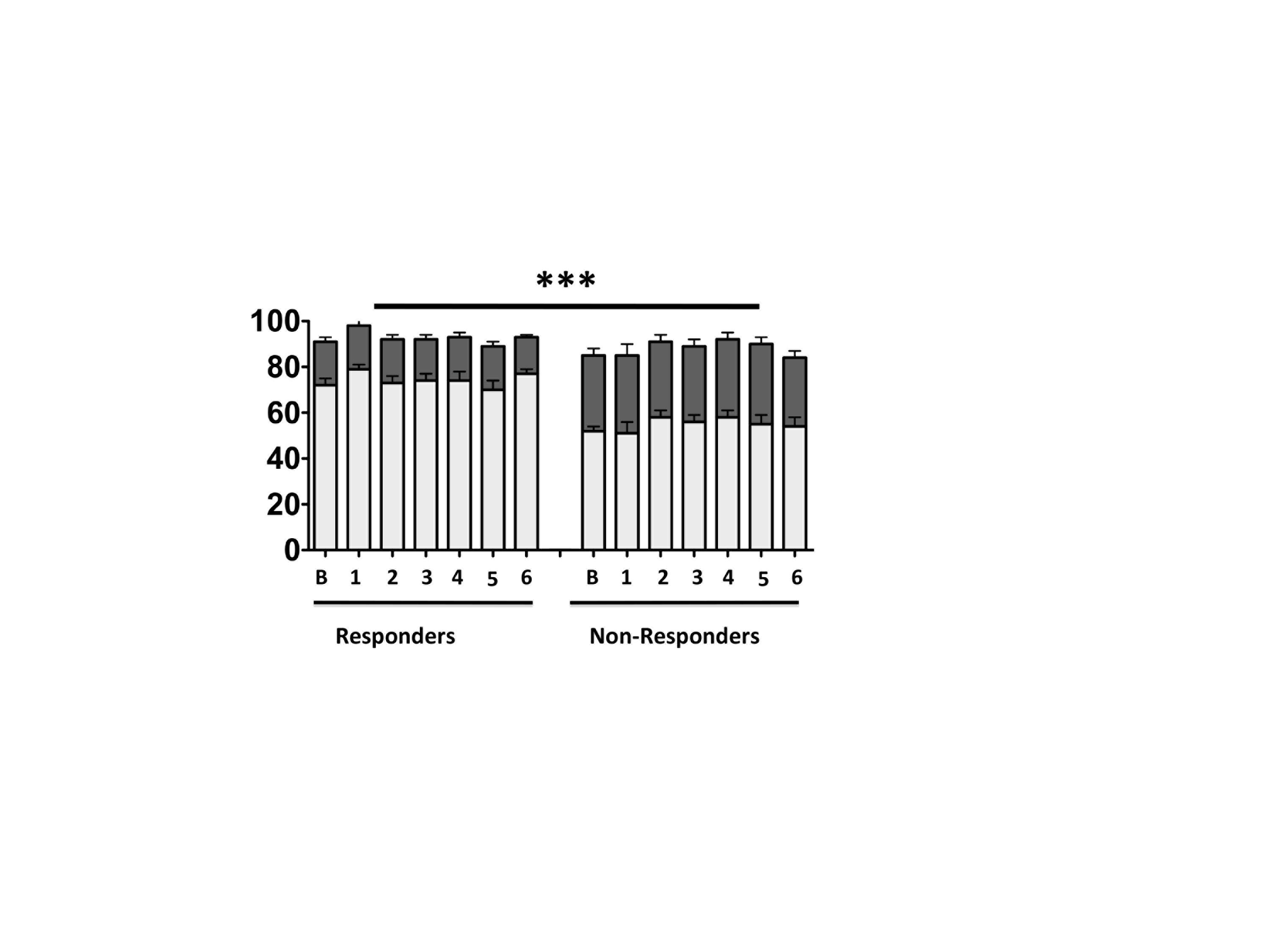

Supplement: Figure S2 — CD3 T cells after tremelimumab exposure. Percentage of CD3+CD4+ (light gray) and CD3+CD8+ (dark gray) T cells before (B) and at different time-points of CTLA4 dosing in the tremelimumab as single agent treatment (***p<0.001). (TIF) [file pone.0076829.s002.tif]

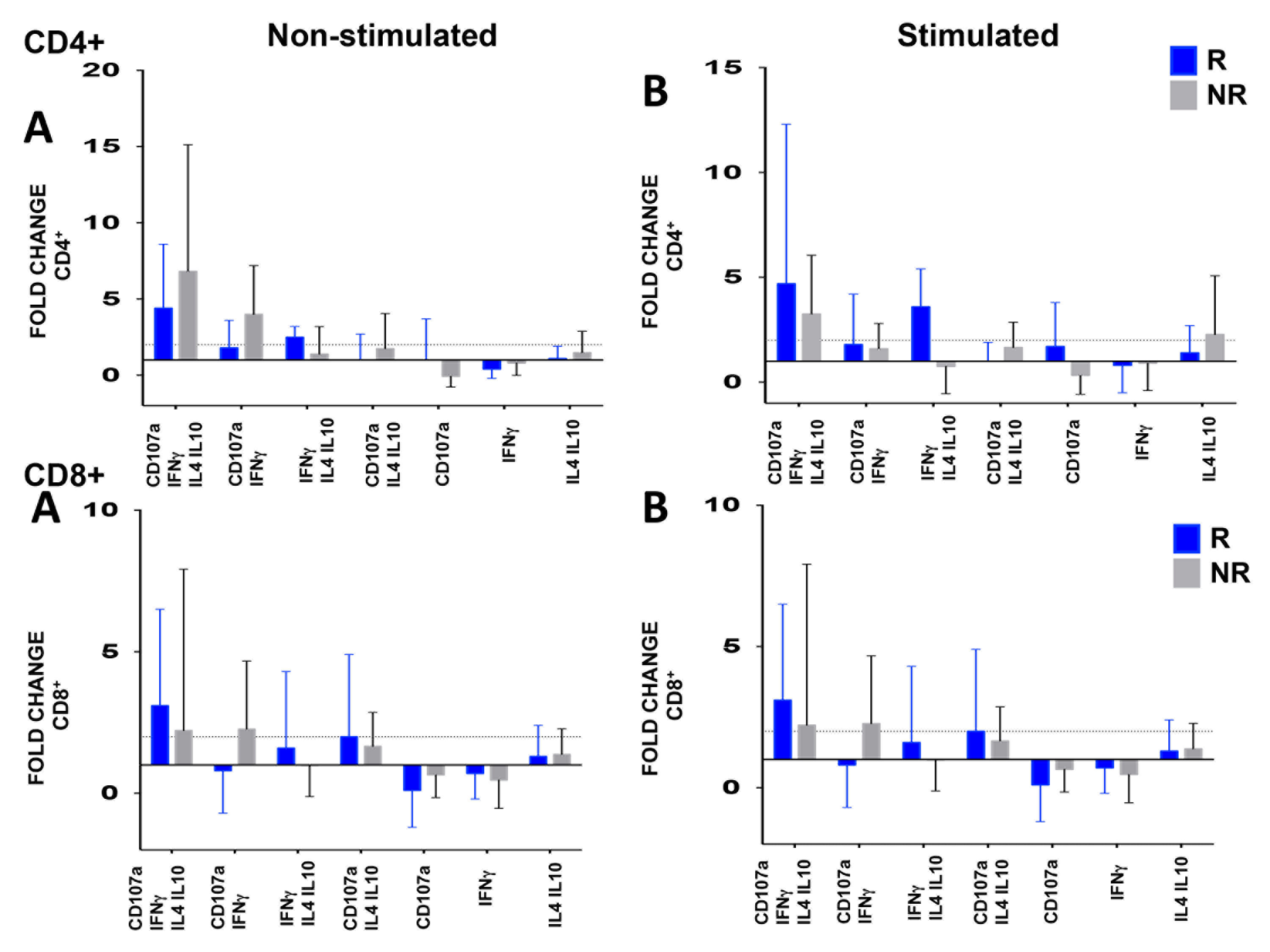

Supplement: Figure S3 — Functionality of T subsets after tremelimumab plus Mart-1/DC treatment. Intracellular staining of IFNγ, IL4, IL10 and CD107a in iNKT cells after tremelimumab plus Mart-1/DC treatment between responders (blue) and non-responders (grey) were measured in PBMC stimulated with OKT-3 plus IL2 for six hours. Y axis displayed fold change of CD4+- and CD8+- T cells after treatment. X axis showed the different cytokines expressed by the cells. Dot line showed two fold change with respect to baseline, and any bar over the two fold is considered a change. (TIF) [file pone.0076829.s003.tif]
