# Supplementary material for: Natural Killer T Cells in Advanced Melanoma Patients Treated with Tremelimumab
Source: PLoS One. 2013 Oct 22;8(10):e76829. doi: 10.1371/journal.pone.0076829 (PMC3805549; doi:10.1371/journal.pone.0076829)
Supplement: Table S1 — Patient characteristics. (DOCX) [file pone.0076829.s004.docx]

Table S 1: Patient characteristics

|  |  | **Patients study** | |
| --- | --- | --- | --- |
|  |  | **NRA** | **GA** |
| Number of patients |  | 8 | 21 |
| Mean age (range)   - responders - non-responders |  | 50 (34-57)  52 (48-57)  49 (34-57) | 52 (27-81)  59 (49-79)  56 (27-81) |
| Female/male   - responders - non-responders |  | 3/5  1/3  2/2 | 4/17  3/0  1/17 |
| Stage | IIIc | 1 | 3 |
|  | M1a | 1 | 2 |
|  | M1b | 3 | 2 |
|  | M1c | 2 | 14 |
| Prior therapy | Surgery only | 1 | 13 |
|  | Immunotherapy only | 3 | 3 |
|  | Chemotherapy only | 2 | 3 |
|  | Immunotherapy and chemotherapy | 1 | 2 |
| Tremelimumab dose | 3 mg/kg qmo months | 2 |  |
|  | 10 mg/kg q3 months | 4 | 0 |
|  | 15 mg/kg q3 months | 2 | 21 |
| MART-1/DC |  | 6 | 0 |
| Toxicities | Grade 2 pruritus | 0 | 2 |
|  | Grade 2 diarrhea | 0 | 3 |
|  | Grade 2 hypophysitis | 0 | 1 |
|  | Grade 3 colitis | 0 | 2 |
| Response | Partial response | 1 | - |
|  | Complete response | 3 | 3 |
