# Supplementary material for: Natural Killer T Cells in Advanced Melanoma Patients Treated with Tremelimumab
Source: PLoS One. 2013 Oct 22;8(10):e76829. doi: 10.1371/journal.pone.0076829 (PMC3805549; doi:10.1371/journal.pone.0076829)
Supplement: Table S2 — Antibody combinations for multicolor surface immune phenotyping of NRA and GA patients. (PDF) [file pone.0076829.s005.pdf]

Supplementary Table 2: Antibody combinations for multicolor surface immune phenotyping of NRA and GA patients.

In parenthesis the clone used.

**Fluorochrome**	**Antibody**			
	**Tube 1**	**Tube 2**	**Tube 3**	**GA Study**
7AAD	7AAD**			
Ax700	CD3 (UCHT1)*			
Pacific Blue	CD8 (3B5)***			
Pacific Orange	CD4 (S3.5)***			
FITC	TCRVβ11 (C21)**			
PE	TCRVα24 (C15)**			
ECD	HLA-DR (Immu-357)**	CD62L (DREG56)**	CD45RA (2H4LDHIILDB9)**	CD45RA (2H4LDHIILDB9)**
Ax647/ APC	CD279 [PD1] (MIH4)*	CD137 (4B4-1)*	CD56 (B159)*	CCR7 (3D12)*
APC-Cy7	CD25 (M-A251)*	CCR5 (2D7/CCR5)*	CD27 (M-T271)*	-

*BDBioscience; **BeckmanCoulter; ***invitrogen; Ax= AlexaFluor; ECD= PE-Texas Red; FTIC= Fluorescein isothiocyanate; APC= Allophycocyanin; PE= Phycoerythrin; Cy= cyanine; TCRV= T Cell Receptor Variable.
