# Supplementary material for: Natural Killer T Cells in Advanced Melanoma Patients Treated with Tremelimumab
Source: PLoS One. 2013 Oct 22;8(10):e76829. doi: 10.1371/journal.pone.0076829 (PMC3805549; doi:10.1371/journal.pone.0076829)
Supplement: Table S3 — Antibody combinations for ICS of NRA patients. In parenthesis the clone used. (PDF) [file pone.0076829.s006.pdf]

Table S 3: Antibody combinations for ICS of NRA patients. In parenthesis the clone used.

Fluorochrome	Antibody (clone)
7AAD	7AAD**
Ax700	CD3 (UCHT1)*
Pacific Blue	CD8 (3B5)***
Pacific Orange	CD4 (S3.5)***
FITC	TCRVβ11 (C21)**
PE	TCRVα24 (C15)**
PE-Cy7	IFN-γ (4S.B3)*
APC	IL4 (8D4-8) * & IL10 (JES3-19F1)*
APC-H7	CD107a (H4A3)*

*BDBioscience; **BeckmanCoulter; ***invitrogen;; FTIC= Fluorescein isothiocyanate; APC= Allophycocyanin; PE= Phycoerythrin; TCRV= T Cell Receptor Variable.
